# Fecal transplant from vaginally seeded infants decreases intraabdominal adiposity in mice

**DOI:** 10.1080/19490976.2024.2353394

**Published:** 2024-05-14

**Authors:** Sivaranjani Namasivayam, Curtis Tilves, Hua Ding, Shaoguang Wu, Jada C Domingue, Camilo Ruiz-Bedoya, Ankit Shah, Eric Bohrnsen, Benjamin Schwarz, Mickayla Bacorn, Qing Chen, Shira Levy, Maria Gloria Dominguez Bello, Sanjay K Jain, Cynthia L Sears, Noel T Mueller, Suchitra K Hourigan

**Affiliations:** a Clinical Microbiome Unit (CMU), Laboratory of Host Immunity and Microbiome, National Institute of Allergy and Infectious Diseases, National Institutes of Health, Bethesda, MD, USA; b Department of Epidemiology, Johns Hopkins University Bloomberg School of Public Health, Baltimore, MD, USA; c Department of Epidemiology, Welch Center for Prevention, Epidemiology and Clinical Research, Baltimore, MD, USA; d Division of Infectious Diseases, Department of Medicine, Johns Hopkins University School of Medicine, Baltimore, MD, USA; e Center for Infection and Inflammation Imaging Research, Department of Pediatrics, Johns Hopkins University School of Medicine, Baltimore, MD, USA; f Inova Health System, Inova Women’s Hospital, Falls Church, VA, USA; g Research Technologies Branch, Rocky Mountain Laboratories, National Institute of Allergy and Infectious Diseases; National Institutes of Health, Hamilton, MT, USA; h Departments of Biochemistry and Microbiology, Rutgers University, New Brunswick, NJ, USA; i Humans and the microbiome program, Canadian Institute for Advanced Research (CIFAR), Toronto, ON, Canada; j Division of Pediatric Gastroenterology, Department of Pediatrics, Johns Hopkins University School of Medicine, Baltimore, MD, USA

**Keywords:** Vaginal seeding, vaginal microbiome transfer, cesarean section, microbiome, microbiota, metabolites, neonate, adiposity, metabolism

## Abstract

Exposing C-section infants to the maternal vaginal microbiome, coined “vaginal seeding”, partially restores microbial colonization. However, whether vaginal seeding decreases metabolic disease risk is unknown. Therefore, we assessed the effect of vaginal seeding of human infants on adiposity in a murine model. Germ-free mice were colonized with transitional stool from human infants who received vaginal seeding or control (placebo) seeding in a double-blind randomized trial. There was a reduction in intraabdominal adipose tissue (IAAT) volume in male mice that received stool from vaginally seeded infants compared to control infants. Higher levels of isoleucine and lower levels of nucleic acid metabolites were observed in controls and correlated with increased IAAT. This suggests that early changes in the gut microbiome and metabolome caused by vaginal seeding have a positive impact on metabolic health.

## GRAPHICAL ABSTRACT

**Figure:**
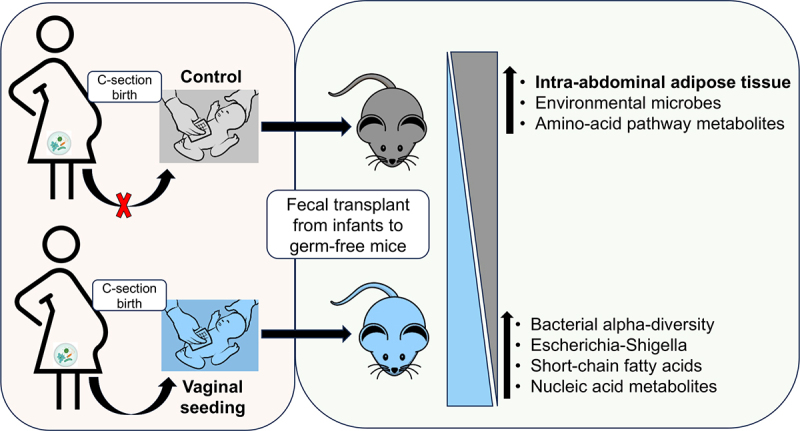

## Introduction

Initial microbial colonization of an infant at birth plays a critical role in metabolic and immune system development.^1–3^ Long-lasting metabolic, immune, and inflammatory consequences have been shown to occur when the timing and order of microbial colonization are disturbed, both in murine models and humans.^4–7^

Cesarean section (C-section) delivery is often necessary and lifesaving for mothers and infants but also disturbs the early life microbial colonization of an infant.^8^ Infants delivered by C-section bypass the microbial exposure from maternal vaginal and perineal sources that they would have otherwise received in a vaginal delivery (VD). Numerous studies have demonstrated differences in early life microbiome acquisition and gut microbiome development in infancy and later into childhood between those delivered by C-section and VD.^9–12^ Moreover, epidemiological studies have shown an association between C-section delivery and several metabolic and inflammatory diseases.^13^ There is evidence from several meta-analyses that C-section delivery is associated with an increased risk of being overweight and obesity, even after controlling for potential confounding factors.^14–17^ The gut microbiome may in part mediate the relationship between C-section and these adverse health outcomes. Evidence to suggest this includes a large longitudinal birth cohort study with repeated microbiome measures, and murine models showing transfer of these disease phenotypes with fecal microbiota transplantation.^4,7^

Wiping down an infant immediately after birth with their own mother’s vaginal fluid, coined “vaginal seeding” (VS), is a means to transfer to the newborn the vaginal microbiome that they do not receive in a C-section delivery. VS was shown in observational studies, and more recently in randomized controlled trials, to allow maternal bacterial engraftment in multiple body sites of a C-section delivered infant and to change the trajectory of the microbiome development closer toward a VD infant.^18–21^ It is currently unknown whether this restoration of the microbiome by VS decreases the risk of C-section associated metabolic and inflammatory diseases.^22,23^ Although there are current blinded randomized controlled clinical trials underway to assess whether VS can decrease the risk of being overweight and obese (ClinicalTrials.gov Identifier: NCT03298334), these are several years from completion due to 1) the large number of subjects needed to power the analyses and 2) follow-up needed over several years to have meaningful adiposity outcomes. Therefore, we aimed to assess the effect of VS of human infants on adiposity in an experimentally controlled murine model.

## Results

### Human subjects

**Figure 1.**
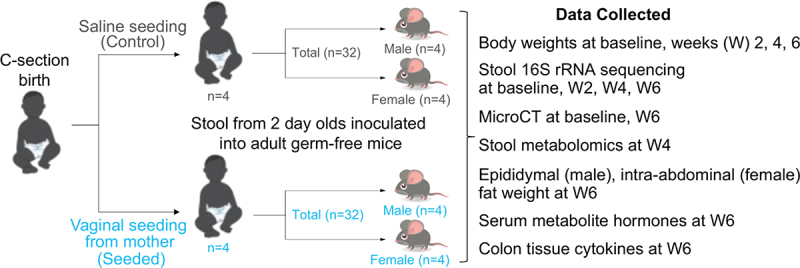
Schematic of experimental design. The transitional stool of each control and vaginally seeded infant was tested in 8 germ-free mice (4 female and 4 male mice).

Eight human infants who were enrolled in an ongoing double-blind randomized controlled clinical trial of VS (see methods for details of clinical trial) were selected. A total of 4 infants had received VS and 4 had received control seeding (placebo with sterile saline). Infants in the VS group and control group were balanced for infant sex, maternal body mass index category, maternal race and ethnicity and whether they received breast milk prior to day 2 of life (Supplemental Table S1). Stool from day 2 of life (i.e. transistional stool) from 4 VS and 4 control group infants was used to orally inoculate germ-free mice. Stool from one VS and one control infant were paired to create 4 different groups and the FMT experiments were performed in 4 waves. For each of the 4 groups, stool samples from the VS and control infant was inoculated into 4 male and 4 female mice each and various data points were collected for subsequent analyses (Figure 1). Transitional stool was chosen for inoculation as this already shows differences by delivery mode and by VS in infants, and contains microbiota with pioneering colonizers (the first set of microbes to colonize an infant).^20,24^

### Adiposity measures between treatment arms

**Figure 2.**
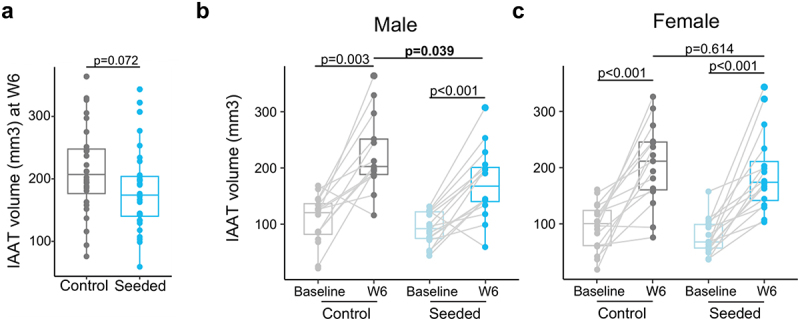
Differences in intraabdominal tissue volume 6 weeks post-inoculation (W6) in mice following fecal transplant from vaginally seeded and control infants: a) all mice b) male mice c) female mice. Statistical significance was assessed by unpaired two-sample Student’s t test between the seeded group and the control group (a-c) and by paired Student’s t test between baseline and week 6 (W6) within each group (b-c).

Computerized tomography (CT) scans obtained pre-inoculation and 6 weeks post-inoculation (W6) were compared between treatment arms (VS versus control). The intraabdominal adipose tissue (IAAT) volume at W6 was lower in the mice that received stool from VS infants compared with control infants; this reached statistical significance for male mice (mean 225.8 vs 172.5 mm^3^, *p* = 0.039) but not female mice (mean 204.7 vs 191.6 mm^3^, *p* = .614) (Figure 2). Baseline pre-inoculation IAAT volumes did not differ between treatment arms. There was no difference in subcutaneous adipose tissue volume between treatment arms at baseline, post-inoculation or by sex.

Overall body weights at W6 post-inoculation and weight gain trajectories post-inoculation between treatment arms were not significantly different in either sex. Epididymal fat tissue weight (males) and abdominal cavity fat weight (females) trended toward being lower in the VS group, but comparisons did not meet statistical significance (Supplemental Figure S1).

### Microbiome measures between treatment arms

16S ribosomal (r) RNA gene sequencing analysis was performed on stool from the mice at week 4 (W4) and W6 post-inoculation and also of the inoculation material. Given the differences in IAAT volumes by sex, microbiome metrics were also assessed by sex as well as together.

**Figure 3.**
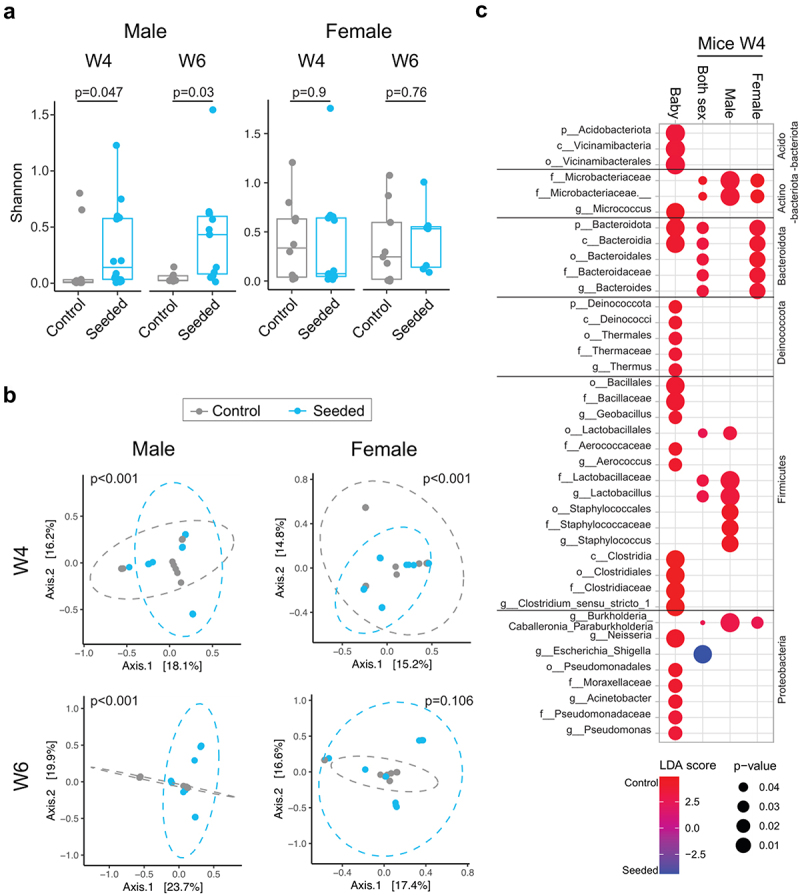
Differences in microbiome metrics at week 4 (W4) and week 6 (W6) post-inoculation in mice following fecal transplant from vaginally seeded and control infants: (a) Alpha diversity estimates calculated using Shannon index at W4 and W6 post-inoculation for male and female mice. Statistical significance between two groups was calculated using Mann Whitney U-test and significance values are indicated. b) Principal component plots of Bray Curtis dissimilarity metrices colored by inoculation group are shown for both timepoints for male and female mice. Statistical significance was calculated using Adonis with 999 permutations. c) LefSe analysis was performed to identify differentially abundant taxa for each pairwise comparison indicated along the x-axis and displayed as a dot plot. Colors representing the differential abundance as defined by the LDA score and significance values are as shown in the key.

Alpha diversity (Shannon index, which provides information on richness and evenness) was higher in the VS group compared with the control group in males at W4 (*p* = .047) and W6 (*p* = .03) but not in females (Figure 3a). Beta diversity (Bray – Curtis dissimilarity) was different between treatment groups in males at W4 (*p* < .001) and at W6 (*p* < .001) and in females at W4 (*p* < .001) (Figure 3b). In addition to the treatment arms being different from each other overall, the control group clustered more closely together whereas the VS group was more dispersed, suggesting more variation in microbiome composition in the latter group (Figure 3b).

Several significantly differentially abundant taxa were identified between treatment arms (Figure 3c). When both sexes were assessed together and separately, there were higher levels of taxa from the Microbacteriaceae family in the control group compared to the VS group. *Staphylococcus* was also higher in the control group, but only in male mice. With both sexes together, the genus *Escherichia-Shigella* was higher in the VS group.

Unexpectedly *Lactobacillus* and *Bacteroidetes* were higher in mice inoculated with stool from the control group, however this was driven by just a few mice with low read counts (Supplemental Figure S2). Of note, different taxa were differentially abundant by treatment group in the inocula versus the murine stool samples, consistent with differential selection of certain human bacteria in the murine gut environment (Figure 3c).^25^

### Stool metabolites between treatment arms

**Figure 4.**
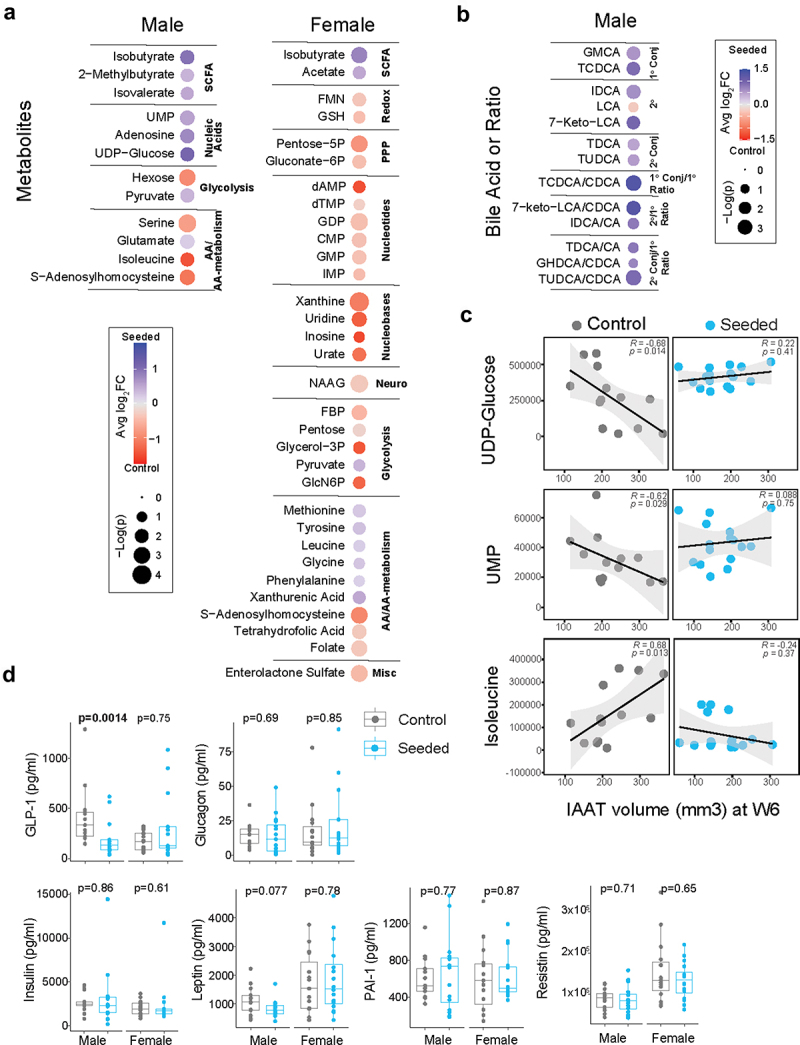
Differences in stool metabolites of mice at week 4 post-inoculation following fecal transplant from vaginally seeded and control infants. (a) Metabolites that display significant difference in abundance between groups as calculated by average fold change are shown for male and female mice. Direction of fold-change and significance values are as represented in the key. Only metabolite differences that pass a cutoff for 20% FDR are displayed. (b) Primary and secondary conjugated bile acids and their ratios that pass a cutoff for 20% FDR are displayed. (c) Spearman’s’s correlation analysis was performed between differentially abundant metabolites/bile acids and IAAT volume for male mice inoculated with stool from vaginally seeded or control infants and correlations found to be statistically significant are displayed. (d) Differences in serum metabolic hormones at week 6 (W6) post-inoculation following fecal transplant from vaginally seeded and control infants. Statistical significance between two groups was calculated using Mann Whitney U-test and significance values are indicated.

Targeted assays (see methods for details) were used on W4 stool; W4 stool was chosen as there was enough stool volume at this time point to generate both metabolite and microbiota data for direct comparative analysis. Several categories of stool metabolites were significantly different between treatment arms (Figure 4a). In both sexes multiple short chain fatty acids (SCFAs) were increased in the VS group with 2-methylbutyrate and isovalerate being more affected in male mice, acetate being most affected in females, and isobutyrate being elevated in both sexes. With respect to carbohydrate metabolism, pyruvate was elevated in both sexes and measured pools of free sugars decreased (pentose in females and hexose in males) in the VS group in comparison to controls. A decrease in S-adenosylhomocysteine with VS was also observed in both sexes. When looking at nucleic acid metabolites, increased uridine-related metabolites (uridine diphosphate-glucose and uridine monophosphate) and adenosine were observed in the VS group (versus C-section group) in male mice only. Additionally, isoleucine, which is involved in amino acid metabolism, was decreased in the VS group compared to the control group.

A mixture of conjugated primary and secondary bile acids were increased in stool from VS male mice compare to control males. Increases in primary-to-secondary bile acid conversion were evident in isodeoxycholic acid to cholic acid, 7-ketolithocholic acid to chenodeoxycholic acid and tauroursodeoxycholic acid to chenodeoxycholic acid (Figure 4b). Notably, inoculation with VS stool did not significantly decrease any bile acids or bile acid conversion ratios in male mice. There were no significantly different bile acids between VS and control mice in females. A correlation analysis between microbiota and metabolites found a negative correlation between *Staphylococcus* and hexose in the seeded group that was driven by a few individuals; otherwise, significant correlations were not found following correction for multiple testing.

To identify metabolites and microbiota that may mediate the effect of VS stool on IAAT in male mice, microbiota and metabolites that were different by treatment arms were levels were examined with respect to IAAT volume by Spearman’s correlation analysis. There were no significant correlations between individual bacterial taxa and IAAT volume. However, higher levels of isoleucine and lower levels of nucleic acid metabolites (uridine diphosphate-glucose and uridine monophosphate) significantly correlated with increased IAAT in the control group (Figure 4c).

### Serum metabolic hormones between treatment arms

Hormones known to be associated with metabolism control were assessed in the serum at W6.^26^ Glucagon-like Peptide-1 (GLP-1) was lower in the VS group compared with controls in male mice (*p* = .001) but not in females (Figure 4d). Leptin also trended toward being lower in the VS group compared with controls in male mice (*p* = .077). No differences were seen between treatment groups in glucagon, insulin, plasminogen activator inhibitor-1 (PAI-1), or resistin in either sex (Figure 4d). GLP-1 did not correlate with IAAT volume in male mice. However, leptin levels positively correlated with IAAT volume in control male mice (*p* = .024) (Supplementary Figure S3).

### Colon cytokine measures between treatment arms

Specific cytokines known to be associated with inflammation, obesity and the gut microbiome were assessed in colon tissue at W6.^27^ Tumor Necrosis Factor alpha (TNF-α) trended toward being higher in the VS group compared with controls in male mice (*p* = .068) but not in females (Supplemental Figure S4). There were no differences in interferon gamma (IFN-γ) and interleukin 17A (IL-17A) between treatment groups in either sex.

## Discussion

In our experimental murine study, fecal transplant form C-section delivered infants who received VS, compared with fecal transplant from C-section delivered infants who received control seeding, decreased intraabdominal adiposity in male mice. This pilot evidence suggests that early life gut microbiome and metabolome changes from VS may have a positive impact on metabolic health.

C-section delivery has been associated with both higher peripheral and central adiposity in offspring later in life.^28^ Compared to peripheral adiposity, intraabdominal adiposity is more strongly associated with an adverse metabolic risk profile, including insulin resistance, metabolic syndrome and cardiovascular disease.^29,30^ Therefore, it was promising to see a reduction in IAAT following fecal transplant from VS human infants suggesting that VS may have metabolic health benefit. However, this effect was only significant in male mice which is consistent with variability in the extent of phenotype emergence seen by sex in other murine models of microbial colonization.^4,31^ Behavioral, metabolic and hormonal signaling differences have been identified as important drivers of sex-based differences in murine metabolic studies and the mechanisms of which are only beginning to be elucidated.^32–34^ In humans, the risk of C-section delivery on offspring adiposity has not been shown to differ by sex in the few studies in which it is reported.^14,16^ This reduction seen in IAAT also emphasizes that in human trials assessing the effect of VS on adiposity, attention may need to be given to measures of intraabdominal adiposity in addition to more standard anthropometrics such as body mass index.

Clear differences were seen in the stool microbiota in mice following fecal transplant from VS versus control infants. In the control group, there was a predominance of *Staphylococcus* (in males only), a common skin bacterial taxon, and Microbacteriaceae (both sexes), typically considered environmental taxa, compared with mice that received stool from VS infants. C-section delivered infants are more commonly colonized with skin and environmental bacteria compared to vaginally delivered infants, with VS being partially corrective of this.^9,19,35,36^ In mice that received stool from VS infants, there were higher levels of *Escherichia-Shigella*, presumably from a maternal perineal source. An early life bloom in Proteobacteria, including *Escherichia sp*, with a subsequent decrease over infancy, is thought to be an important contributor to immune education in early life as opposed to later in life where it is often considered proinflammatory.^37^ There is also evidence that very early life microbiota-driven TNF-α can drive certain aspects of immune maturation.^38^ Although not statistically significant, increased colonic TNF-α was seen in VS mice compared to controls. It is also important to note that there was more variation in the composition of the microbiome within the VS group, as compared to the control group that clustered more closely together. Heterogeneity is seen in the pregnant human vaginal microbiome composition and between infants who receive VS in the overarching clinical trial.^20^ It will be important to investigate whether this heterogeneity in maternal vaginal microbiota leads to differential emergence of infant phenotypes in subsequent larger studies.

Many stool metabolites also differed between treatment arms. There was a decrease in several SCFAs (both sexes) and certain nucleic acid metabolites (males) in the control group compared to VS group. Of these nucleic acid metabolites in our study, decreased uridine-related metabolites (uridine diphosphate-glucose and uridine monophosphate) correlated with increased IAAT in controls. Higher uridine levels have been shown to be protective against obesity in other murine models.^39,40^ Isoleucine positively correlated with IAAT in controls in our study. Prior studies have shown that higher levels of isoleucine correlate with increased obesity in children and adolescents and that reduced isoleucine promotes metabolic health in murine models^41,42^ As such, these metabolites may represent functional pathways by which VS may help reduce IAAT and warrant further investigation. Furthermore, a decrease in S-adenosylhomocysteine with VS was observed in both sexes, which is implicated in quorum sensing mediated biofilm formation.^43,44^ Given that inhibition of S-adenosylhomocysteine has been implicated in attenuated virulence of bacteria due to reduced biofilm formation, it could be speculated that these decreased levels observed in VS mice may be beneficial for the host.

When looking at serum hormones involved in the control of metabolism, GLP-1 was lower in the VS group compared to the control group in male mice. After food intake, GLP-1 inhibits gastrointestinal motility leading to increased food retention, stimulates insulin secretion, and inhibits glucagon secretion thus regulating postprandial glucose control.^45^ Therefore, we theorize that the VS male mice were in a more physiologically “normal” state requiring lower GLP-1 levels compared to the control male mice that had a higher IAAT volume. Leptin levels trended toward being lower in VS mice compared to controls in males. Leptin is predominantly produced by adipose tissue and regulates food intake and body mass.^46^ Therefore, it is not surprising that leptin was positively correlated with IAAT volume in control male mice. These intriguing observations of increased GLP-1 and leptin levels in control males compared with VS males indicate further investigation is needed using longer term murine obesity models to identify physiological significance.

To our knowledge this is the first study to show that fecal transplant from VS infants is sufficient to cause changes in the early life gut microbiome and metabolome and reduce IAAT volume, thus having a positive impact on metabolic health. Nevertheless, there are several limitations. Firstly, transitional stool was used for inoculation as this already shows differences by delivery mode and by VS in infants, and contains microbiota with pioneering colonizers.^20,24^ However, the bacterial load in transitional stool is very low and so this limited the ability to analyze the microbiome at early timepoints after inoculation and therefore future studies involving fecal transplant with transitional stool might consider performing multiple inoculations to achieve higher levels of bacterial transfer. In addition, as transitional stool from day 2 was used, some effects of environmental exposure i.e. not just exposure from vaginal seeding, may also be present but this should be balanced in both arms. Second, the mice were inoculated at 8 weeks of age for stability which does not fully represent the physiological situation of VS of human infants in which newborns receive the seeding and thus changes that occur only in early life developmental window may have been missed.^47^ Future studies should investigate whether a more pronounced effect may be seen if neonatal mice are inoculated. Thirdly, the mice did not undergo any stressors such as a high fat diet which may have produced a more prominent phenotype; however, despite this a difference in IAAT between treatment arms in male mice was seen. It is also possible that the genetic background of mice may influence the results. Finally, this study only examines the effect of pioneering colonizers from VS on adiposity and not subsequent microbiome evolution due to diet or other environmental influences which may also have an important role in metabolic health. Further investigation is warranted using stool from infants in both arms at later time points to address this. Additionally, future studies investigating further metabolic outcomes such as adipocyte size, and immune and inflammatory development, both local to the gut and systemically, may prove fruitful.

## Methods

### Experimental model and subject details

#### Human study subjects

Pregnant women who were scheduled for an elective C-section ≥37 weeks gestation were recruited and written informed consent was obtained. Stringent inclusion and exclusion criteria were used to ensure the least risk of maternal to infant transmission of infection and the exclusion of maternal health conditions associated with dysbiosis of the vaginal microbiome. For full details of inclusion and exclusion criteria see https://clinicaltrials.gov/ct2/show/NCT03298334.

### Human study procedure: randomization and vaginal seeding

Full details of the human study have been previously described.^20^ In brief, on the day of the scheduled C-section, a blinded team member inserted a gauze moistened with sterile saline into the mother’s vagina. The gauze was incubated in the vagina for approximately 1 hour and then removed and placed into a sterile container prior to the mother receiving perioperative antibiotics. A control gauze was also made by identical preparation of the gauze but without insertion into the mother’s vagina. An unblinded team member then randomized the mother to either the VS arm or the control arm. Randomization occurred in a 1:1 ratio and was also stratified to 3 pre-pregnancy BMI strata: normal weight, overweight and obese.

In the operating room, after C-section delivery of the infant and prior to the infant having skin–skin contact with their mother, the infant was wiped down with the VS gauze or the control gauze by a blinded team member. The gauze was wiped first over the infant’s mouth, followed by the face and the rest of the body in a standardized manner as previously described, typically just after the 1-minute APRGAR score was taken.^18–20^ For this reported study, 8 infants were selected, 4 who received VS and 4 who received the control gauze. Infants were selected before unblinding; select members of the team conducting the analysis were only unblinded after final analysis. Infants were selected sequentially if they successfully underwent the VS or control seeding process and had sufficient Day 2 stool available for transfer into mice.

### Human data and samples

Detailed data on mothers was collected including demographics, medical history, anthropometrics and medication use including antibiotics during pregnancy. Data collected for the infants included demographics, method of first feeding and subsequent feedings, medication use and adverse events. Data was collected and managed using the REDCap electronic data capture tools hosted at the Inova Health System.

For this reported study, day 2 of life stool from the infants was used. Stool samples were stored in 2 ml Eppendorf safe-lock tubes (cat# 0030123620) at −80°C until the animal study procedures.

### Mice

Germ-free mice were housed in the Germ-Free Mice Core at Johns Hopkins University School of Public Health. The mice were bred in sterile bubble isolators (Classical Biological Control #50602424122) with unlimited sterile diet (Lab Diet #5k67) and double autoclaved tap water. The mice were screened monthly by microbiological culture and 16s rRNA PCR of fecal pellets to ensure their germ-free status.

When a cohort of 8 male or female mice at 8 weeks-old age became available, mice were transferred to individual sterile isocages (4 mice/cage) (Allentown, Sentry SPP) where they were maintained throughout the experiment. To work with the mice, isocages were sprayed on the outside with Peridox (VWR 19-033-460/CR85335) that was allowed to sit for 5 minutes, prior to opening the cages inside a biosafety cabinet.

Thirty-two male and 32 female germ-free C57BL/6 mice were used for 8 individual experiments. 3 male mice that developed severe wounds and one female mouse that became ill before the second CT scan were euthanized.

## Method details

### Human stool inoculum

Stool from day 2 of life collected from human infants stored at −80°C was used as the mouse inoculum. A 4% stool slurry in PBS was made in an anaerobic chamber. Each mouse was inoculated with 100 μl of slurry by 18 G stain steel gavage needle at 8 weeks of age, after their first (baseline) CT scan. The inocula were set up as 4 pairs (each pair with one stool from a VS infant and one stool from an infant swabbed with the control gauze). Four female and four male mice received each inoculum peroral.

### Mouse data and samples

#### CT scans

In vivo high-resolution CT imaging (nanoScan PET/CT, Mediso USA, LLC) was performed at the Center for Infection and Inflammation Imaging Research (Ci3R), Johns Hopkins University School of Medicine (SP20M144) at baseline (pre-inoculation) and at W6. Imaging beds and the anesthesia chamber were sanitized with 10% bleach solution and UV light overnight to maintain sterility. The induction anesthesia was performed by a nose cone filtered through a 0.22 μm PTFE filter with 5% isoflurane rate for 2–5 minutes until breath rates slowed visibly.^48,49^ Eyes were protected with ointment (Artificial Tears Ophthalmic Ointment 83%/15% 3.5 gram PF sterile, Henry Schein Inc. Cat# 1380963). Upon transfer to the imaging bed, the mouse was placed prone in the center of the bed with both legs fully extended. The isoflurane level was adjusted to 1.0–1.5% to keep the animal anesthetized to avoid artifacts. Mice were imaged from vertebrae L1 to L6 using 50kVp x-ray voltage. CT images were reconstructed to 40 μm^3^ sized voxels. Subcutaneous fat and intraabdominal fat were quantified using fixed thresholding of −500 to −120 Hounsfield Units.^50,51^

#### Other measures

Body weights and stool samples were obtained at baseline (pre-inoculation) and then every 2 weeks.

#### Tissue harvest and blood collection

At W6, immediately after the second CT scan, the mice were euthanized for tissue harvest. Blood was collected in BD Microtainer tubes (cat# 365967) by cardiac puncture, with serum collected on the upper phase of the gel after centrifugation at 2000 (g) for 10 minutes at 4°C (Labnet Prism R). Epididymal (male) fat pads were dissected and weighed. All fat tissue in the abdominal cavity was collected for female mice. The first 1 cm of colon from the anus (with content cleared) was collected. All were snap frozen in liquid nitrogen before storage at −80°C.

### DNA extraction and sequencing

DNA was extracted from stool samples using MagAttract PowerMicrobiome DNA/RNA Kit (QIAGEN) following the manufacturer’s guideline for automated workflow (epMotion5073). DNA was quantified using NANODrop 8000 Thermo Scientific. The V4 region (the current gold standard for stool microbiome sequencing) of the 16S rRNA gene was amplified using the primers (5’-TCGTCGGCAGCGTCAGATGTGTATAAGAGACAGGTGCCAGCMGCCGCGGTA-3’ and 5’-GTCTCGTGGGCTCGGAGATGTGTATAAGAGACAGGGACTACHVGGGTWTCTAAT-3’) and sequenced using the Illumina MiSeq as previously described.^52,53^ For each sequencing run, fecal DNA extracted and combined from 60 different mouse stool pellets, water and a blank template were included as controls.

### Metabolite and lipid sample preparation

For all liquid chromatography mass spectrometry (LCMS) methods LCMS grade solvents were used. To each frozen fecal sample, 400 µL of ice-cold methanol was added. To each methanol-immersed fecal sample, 400 µL of water and 400 µL of chloroform was added. Samples were agitated for 30 minutes at 4°C then centrifuged at 16k ×g for 20 min. 200 µL of the top (aqueous) layer and bottom (organic) layer was collected. The aqueous layer was sub-aliquoted for SCFA derivatization or diluted 5× in 50% methanol in water and prepared for LCMS injection. The organic layer was taken to dryness in a speed vac for 20 minutes at 65°C and 100 mTorr and resuspended in 200 µL 6:1 isopropanol:methanol with 5 µg/mL of butylated hydroxytoluene for bile acid analysis.

#### Short chain fatty acid derivatization

Samples were derivatized with O-benzylhydroxylamine (O-BHA) using previously established protocols with modifications.^54,55^ A reaction buffer was made to 1 M pyridine and 0.5 M hydrochloric acid in water. An aliquot of 35 µL of the original aqueous metabolite extract was taken; 10 µL of 1 M O-BHA in reaction buffer followed by 10 µL of 1 M 1-Ethyl-3-(3-dimethylaminopropyl)carbodiimide in reaction buffer were added to the subaliquot. Samples were allowed to derivatize for 2 hours at room temperature with constant agitation. The reaction was quenched with 50 µL of 0.1% formic acid for 10 min. A volume of 400 µL ethyl acetate was added to the sample. The samples were agitated and centrifuged at 16k ×g for 5 min and 4°C to induce layering. The upper (organic) layer was collected and taken to dryness under vacuum. Samples were resuspended in 300 µL of water for LCMS injection.

#### Liquid chromatography mass spectrometry

Tributylamine and all synthetic molecular references were purchased from Millipore Sigma. LCMS grade water, methanol, isopropanol and acetic acid were purchased through Fisher Scientific. All samples were separated using a Sciex ExionLC™ AC system and measured using a Sciex 5500 QTRAP® or Sciex 6500+ QTRAP® mass spectrometer.

A previously established ion pairing method was utilized for the analysis of the polar metabolites with modification.^56^ Quality control samples were injected after every 10 injections to control for signal stability. A Waters™ Atlantis T3 column (100Å, 3 µm, 3 mm X 100 mm) was used to separate samples with a binary gradient from 5 mM tributylamine, 5 mM acetic acid in 2% isopropanol, 5% methanol, 93% water (v/v) to 100% isopropanol over 15 minutes. Two distinct multiple-reaction monitoring (MRM) pairs in negative mode were used for each metabolite when possible. Heavy labeled standards were not utilized, and relative quantification was performed.

A Waters™ Atlantis dC18 column (100Å, 3 µm, 3 mm X 100 mm) was used to separate O-BHA-derivatized SCFA samples using a 6 min gradient from 5% to 80% B with buffer A as 0.1% formic acid in water and B as 0.1% formic acid in methanol. Short chain fatty acids and central metabolic metabolites were detected using MRMs from previously established methods and identity was confirmed by comparison to derivatized standards.^54,55^

#### Bile acids

Bile acids were separately assayed in all stool samples. Bile acids were separated using a Kinetex® Polar C18 (100Å, 2.6 µm, 3 mm X 100 mm) column. A binary gradient was used consisting of A: 0.01% acetic acid in water and B: 0.01% acetic acid in methanol over a 20 min gradient from 40% to 100% B. Samples were detected in negative mode using previously established MRMs and pseudo-MRMs.^57^ A blank and QC mix were serially injected every 10 injections to ensure instrument stability. Retention times were confirmed using fecal and gallbladder reference samples generated in house as well as injection of Bile Acid SPLASH® (Avanti Polar Lipids, Inc).

All signals were integrated using SciexOS® Software 2.0.0. Signals with greater than 50% missing values were discarded and remaining missing values were replaced with the lowest registered signal value. All signals with a QC coefficient of variance greater than 30% were discarded. Metabolites with multiple MRMs were quantified with the higher signal to noise MRM. Filtered datasets were total sum normalized prior to analysis unless otherwise indicated. The SCFA dataset was stitched to the polar metabolite dataset via common signals for lactate. Single and multi-variate analysis was performed in MarkerView® Software 1.3.1. All univariate comparisons were subjected to a Benjamini–Hochberg cutoff at a false discovery rate as indicated in their respective figures.

### Serum metabolic hormones measurement

Mouse serum concentrations of metabolic hormones were assessed using Bio-Plex Pro mouse diabetes immunoassay kit (Bio Rad, 171F7001M) according to the manufacturer’s protocol and measured using Bio-Plex® 200 System (Bio Rad).

### Gut cytokines

Cytokines, IFN-γ, IL-17A and TNF-α, were assessed in colon tissue homogenates using the respective DuoSet ELISA kit (R&D Systems) according to the manufacturer’s instructions and measured using a BioTek Epoch Microplate Spectrophotometer (Agilent). Total protein was measured by Pierce^TM^ Bradford Assay (ThermoFisher) and cytokine levels were standardized to total protein content.

## Statistics

All anlyses were conducted in a blinded manner and selected members of the team conducting the analysis were only unblinded after final analysis.

### Outcome data

All outcome data were tested for normality. For comparisons between treatment arms, if all data were normal then a parametric students’ t test was used and if data were non-normal a non-parametric test (Mann Whitney U-test or Spearman’s correlation) was used.

#### CT data analysis

Two sample t tests with equal variances were used to compare the mean differences in mouse SAAT and IAAT volumes by treatment arm. The mean differences at the pre-inoculation (baseline) timepoint to assess initial balance by treatment arms and at the 6-week post-inoculation timepoint to assess potential treatment differences were tested. These analyses were performed in the overall treatment group and in sex-stratified cohorts.

#### Other outcome data

Differences in body weight post-inoculation, perigonadal fat weight, serum metabolic hormones and colon tissue cytokines were compared between treatment arms using a Mann Whitney U-test. These analyses were performed in the overall treatment group and in sex-stratified cohorts.

### Microbiota and metabolomics analysis

The sequenced demultiplexed paired-end fastq reads were processed and analyzed using QIIME2 version 2–2022.2.^58^ The DADA2 algorithm, implemented in QIIME2 was used for error modeling and filtering the raw fastq files.^59^ After denoising and chimera removal we obtained a total of 13,001,637 reads from 217 samples. Only 135 out of 217 samples had greater than or equal to 2000 sequencing reads.

Taxonomic classification was performed using Silva database release 138.^60^ The samples were rarefied at a sampling depth of 2,000 reads for alpha diversity analyses. Alpha-diversity was estimated using the Shannon index and statistical significance was assessed using a non-parametric *t* test. Bray-Curtis dissimilarity index was used to estimate beta-diversity and visualized on a two-dimensional principle component plot generated using phyloseq package in R.^61^ Adonis with 999 permutations was used to test the statistical significance of differences in the clustering pattern between the study groups. Linear discriminant analysis (LEfSe) was used to identify differentially abundant taxa in pairwise comparisons.^62^ 16S rRNA sequencing reads for the infant fecal samples transplanted to germ-free mice were obtained from SRA ID 954,027 and processed through the DADA2/QIIME2 and LEfSe pipeline as described above to identify differentially abundant taxa in the inoculum. Taxa with a linear discriminant (LDA) score of >2 and *p* value of < .05 were considered statistically different and these differentially abundant taxa for each pair-wise comparison tested were displayed using ggplot in R.

Metabolomics data was divided by sex due to large sex-dependent shifts in the metabolome. Data was transformed as the log_2_ fold change of each individual mouse back to the corresponding average control set value for that metabolite in that infant pair. The consensus effect of seeding was assessed by comparing seeding to control across all mice within each sex via t-test and imposing a cutoff at a false discovery rate of 20% for all metabolites using the Benjamini–Hochberg method. Bile acids were analyzed based both on abundance as well as ratiometrically based on the conversion of primary to secondary bile acids and the conjugated bile acids compared to the parent primary bile acid.

## Supplementary Material

Supplemental Material

## Data Availability

Sequencing reads are deposited in the Sequence Read Archive (SRA) under submission PRNJA985247, publicly available as of the date of publication. Metabolite data is deposited at https://figshare.com/articles/dataset/Polar_metabolomics_and_Bile_acid_data_for_Namasivayam_2023/23699571. This paper does not report original code.

## Data availibility

**Lead contact**

Further information and requests for resources and reagents should be directed to and will be fulfilled by the lead contact, Suchitra Hourigan (suchitra.hourigan@nih.gov).

**Materials availability**

This study did not generate new unique reagents.

## Study approval

Human subjects in the study were enrolled under the institutional review board approved study (WCG IRB # 1300043) at the Inova Health System in Northern Virginia under the US Food and Drug Administration IND# 18076. Written informed consent was obtained.

Germ-free mice were housed in the Germ-Free Mice Core at Johns Hopkins University School of Public Health under SOP SP20M233.
