# Supplementary material for: Fecal transplant from vaginally seeded infants decreases intraabdominal adiposity in mice
Source: Gut Microbes. 2024 May 14;16(1):2353394. doi: 10.1080/19490976.2024.2353394 (PMC11095576; doi:10.1080/19490976.2024.2353394)
Supplement: Supplemental Material [file KGMI_A_2353394_SM7886.zip › Combined supplemental data R1.docx]

**Supplemental Table 1:** Demographics and clinical characteristics of human subjects between vaginal seeding and control groups.

|  | **Vaginal Seeding Group**  **(n=4)** | **Control group**  **(n=4)** |
| --- | --- | --- |
| **Infant characteristics** |  |  |
| Infant sex female | 2 (50%) | 2 (50%) |
| Infant birth weight mean (range) in  kg | 3.14 (3.00 - 3.71) | 3.43 (2.38 - 3.95) |
| Breast milk only at first feeding | 3 (75%) | 4 (100%) |
| Infant received breast milk prior to  stool sample | 4 (100%) | 4 (100%) |
| Infant received any formula prior  to stool sample | 3 (75%) | 1 (25%) |
| Medication use | 1 (25%)  Dextrose 40% oral gel | 1 (25%)  Zinc oxide |
| Number of infants with adverse events during delivery hospitalization  Adverse events (all non-related) | 3 (75%)   1. Hypoglycemia 2. Facial bruising 3. Slight protrusion near chest | 2 (50%)   1. Hypoglycemia, circumoral cyanosis, diaper rash 2. Bilateral groin with excoriation and bruising, |

1

|  |  | congenital dislocation of  hip (bilateral) |
| --- | --- | --- |
| **Maternal characteristics** |  |  |
| Maternal pre-pregnancy body mass index:  <25 kg/m2  25 - <30 kg/m2  >30 kg/m2 | 3 (75%)  0 (0%)  1 (25%) | 3 (75%)  0 (0%)  1 (25%) |
| Maternal gestational weight gain  mean (range) in kg | 16.75 (9.05 - 31.70) | 14.72 (10.88-19.02) |
| Maternal race:  White or Caucasian Asian | 3 (75%)  1 (25%) | 4 (100%)  0 (0%) |
| Maternal Ethnicity: Not Hispanic or Latino  Unknown | 3 (75%)  1 (25%) | 4 (100%)  0 (0%) |

2

**A**

120


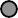

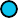


p=0.688 p=0.281 p=0.380

115

% starting weight

110

Male

Control Seeded

**B**

0.8

Male Female

p=0.163

p=0.129

105 0.6

Perigonadal fat weight (g)

100

95

0 2 4 6

Weeks post inoculation

0.4

0.2

120


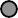

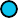


p=0.888 p=0.712 p=0.351

115

% starting weight

Female

0.0

110

105

100

95

0 2 4 6

Weeks post inoculation

Male

Female

Male

Female

0.3

2

0.2

% g_Bacteroides

% g_Lactobacillus

1 0.1


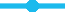

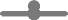

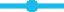

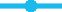

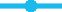

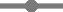
0
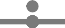
 0.0
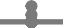


#
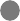
Control

Male


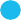
Seeded

2000


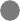

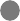

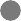

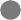

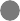

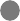

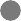

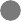

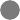

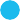

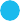

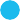

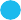

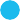

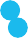


*R* = 0.63

*p* = 0.024

*R* = 0.28

*p* = 0.29

1500

Leptin (pg/ml)

1000

500

0

100 200 300 100 200 300

# IAAT volume (mm3) at W6

Male Female Male Female Male Female

p=0.35 p=0.21

120

p=0.4 p=0.47

125

p=0.068 p=0.67

90 100

IL-17A (pg/mg)

TNF-α (pg/mg)

90

IFN-γ (pg/mg)

75

60


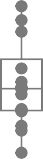
60

50

30

30 25
